# Epigenetic Modulators and Immunotherapy in Malignant Melanoma

**DOI:** 10.32604/or.2026.072349

**Published:** 2026-07-16

**Authors:** Ioannis Anestopoulos, Sotiris Kyriakou, Maria Deligiorgi, Dimitrios T. Trafalis, Sotiris Botaitis, Rodrigo Franco, Aglaia Pappa, Mihalis I. Panayiotidis

**Affiliations:** 1 Department of Cancer Genetics, Therapeutics & Ultrastructural Pathology, the Cyprus Institute of Neurology & Genetics, Nicosia, Cyprus; 2 Laboratory of Pharmacology, Medical School, National & Kapodistrian University of Athens, Athens, Greece; 3 Department of Surgery, School of Medicine, University Hospital, Democritus University of Thrace, Alexandroupolis, Greece; 4 School of Veterinary Medicine & Biomedical Sciences, University of Nebraska-Lincoln, Lincoln, NE, USA; 5 Redox Biology Centre, University of Nebraska-Lincoln, Lincoln, NE, USA; 6 Department of Molecular Biology & Genetics, Democritus University of Thrace, Alexandroupolis, Greece; 7 Department of Comparative Biomedical Sciences, College of Veterinary Medicine, Mississippi State University, Starkville, MS, USA

**Keywords:** Malignant melanoma, epigenetics, DNA methyltransferase inhibitors, Histone Deacetylase inhibitors, immunotherapy, immune checkpoint inhibitors

## Abstract

Despite the use of targeted and/or immune-based therapeutic approaches, mortality rates among melanoma patients are high, mainly due to drug-induced resistance mechanisms. In parallel, alterations of epigenetic mechanisms (e.g., deregulated patterns of DNA methylation, aberrant histone modifications and abnormal expression levels of non-coding RNAs [ncRNAs]) have been associated not only with the pathophysiology of melanoma but also with the resistance against various immunotherapeutic drugs. In this review article, we discuss the involvement of different types of epigenetic mechanisms in melanoma progression. In addition, we report on melanoma’s immune environment and immunosuppressive mechanisms while we highlight the role of immune checkpoint inhibitors (ICIs) as an anti-melanoma therapeutic approach. Moreover, we describe the underlying mechanism(s) by which deregulated epigenetic patterns promote drug resistance against ICIs and how epigenetic drugs (utilized either alone or in combination with various ICIs) can reverse immune resistance. Furthermore, we discuss the major limitations and future directions towards clinical translation of epigenetic drugs, mainly in combination with ICIs. Finally, we state the potential use of emerging technologies (e.g., single-cell transcriptomics and spatial transcriptomics), along with epigenetic priming for improvement of clinical implementation and therapeutic outcomes in melanoma management.

## 1 Background and Therapeutic Approaches in Malignant Melanoma

### 1.1 Risk Factors and Genetic Background

Melanoma is considered the most aggressive skin cancer as it is associated with approximately 80% of all skin cancer-related deaths due to its high metastatic and invasive potential [1,2,3]. In addition, the low 5-year survival rate of the disease (approximately 10–15%) suggests the limited efficacy of current treatment options [4,5]. On the other hand, a number of both environmental and genetic factors have been associated with the onset and progression of melanoma. Specifically, exposure to solar UV radiation is the primary risk factor of melanoma development as 60–70% of malignant melanoma cases are estimated to be caused by prolonged exposure to solar radiation [2,6,7]. In addition, other risk factors include skin color [2], high number of abnormal/dysplastic nevi [8], familial history [9] and immuno-suppression as subject to organ transplantation [10]. At the genetic level, malignant melanoma is characterized by several mutations in genes associated with the onset and/or progression of the disease. For instance, 40–60% of cases are characterized by mutations in the *BRAF* gene localized on chromosome 7, a proto-oncogene encoding a kinase involved in the Rat sarcoma virus protein (Ras), Rapidly accelerated fibrosarcoma kinase (Raf), Mitogen-activated protein kinase (MEK), and Extracellular signal-regulated kinase (ERK)/Mitogen-activated protein kinase (MAPK). Ras/Raf/MEK/ERK(MAPK) signaling pathway [8,11]. Specifically, the *V600E* missense mutation in the *BRAF* gene is one of the most common mutations representing a primary therapeutic target in melanoma management [11,12]. In parallel, activating mutations in the *RAS* gene are also found in 15–20% of melanoma patients (the second most common mutation type) which have been related to increased cellular proliferation and lower survival rates through the abnormal activation of the MAPK cascade [13]. In addition, other important mutations include *K-HRAS*, *p53*, *CDKN2A*, *TERT*, *PTEN* [4] and the recently described *NF1* and *RAC1* genes, among others, ultimately affecting de-regulation of other signaling pathways such as the Janus kinase-signal transducer and activator of transcription (JAK/STAT), Phosphatidylinositol 3-kinase/Protein kinase B (PI3K/Akt), Jun N-terminal kinase (JNK), NOTCH and microphthalmia-associated transcription factor (MITF) [14,15]. These pathways confer to melanoma cells additional properties including: (i) abnormally increased growth rates independent of growth factors, (ii) evasion of apoptotic cell death and immune system surveillance, (iii) increased rates of vascularization and ultimately (iv) increased metastatic and invasive potential [16].

### 1.2 Therapeutic Management

Generally, there are two distinct categories of therapeutic management in melanoma: (i) standard and (ii) targeted therapies [17,18]. While there are currently several standard therapies (e.g., surgery, radiotherapy and chemotherapy) the development of targeted therapies (including immunotherapies) has significantly contributed to the management of melanoma [19,20]. In general, when the tumor is localized, surgery is the main treatment approach while the use of radiation therapy is followed either when localized surgery is not applicable or to minimize recurrence rates after surgical intervention [21]. In parallel, chemotherapeutic agents are usually administrated in advanced stages of the disease and such treatment option was the main approach for several decades. Specifically, Dacarbazine (DITC) although was the first FDA approved drug for the treatment of advanced melanoma, its therapeutic efficacy was rather limited. On the other hand, Temozolomide (TMZ) has been extensively used in treating advanced stages of melanoma and although contributed to a slightly improved median progression-free survival (PFS), compared to DITC, it was shown to exert no significant differences in overall survival rates [22]. Overall, TMZ is considered a good treatment approach in patients with brain metastases under clinical protocols combining surgical intervention and/or radiation therapy [4,23]. Finally, other classes of chemotherapeutic drugs (e.g., cisplatin, carboplatin, docetaxel and paclitaxel) have been also utilized as a second-line treatment option in metastatic patients because of their low effectiveness [23].

The MAPK signaling pathway is implicated in several biological processes including uncontrolled proliferation, metastatic potential and angiogenesis, among others, thus favoring melanoma progression. As such, pharmacological disruption of the above signaling pathway is of paramount importance for the therapeutic management of the disease [24]. In this context, several BRAF inhibitors (BRAFis) have been developed including Dabrafenib, Vemurafenib and Encorafenib, all of which were associated with significant therapeutic efficacy in patients carrying either BRAF^V600E^ or BRAF^V600K^ mutations [25]. However, the development of drug resistance, due to re-activation of the MAPK cascade, led to the development of inhibitors of other components of the MAPK signaling pathway known as MEK inhibitors (MEKis) which included Cobimetinib, Binimetinib and Trametinib [26,27]. Moreover, combinatorial therapeutic protocols involving MEKis [28,29] and BRAFis [30,31] have been used in the clinical practice with increased effectiveness, as opposed to monotherapies.

In recent years, the use of ICIs has further contributed to the management of unresectable and/or metastatic melanoma. Briefly, two types of monoclonal antibodies were approved by FDA including: (i) Ipilimumab [Cytotoxic T Lymphocyte-associated Antigen-4 (CTLA-4) inhibitor] as well as (ii) Nivolumab and Pembrolizumab [programmed cell death protein 1 (PD-1) inhibitors]. Specifically, Ipilimumab inhibits the interaction between receptors and the B7 ligand in the surface of the activated Antigen Presenting cells (APC) thus preventing the inactivation of the T-lymphocytes and ultimately allowing their activity against malignant cells. In comparison, Nivolumab and Pembrolizumab inhibit the interaction between the PD-1 receptor and PD-L1 ligand thus allowing the detection and elimination of cancer cells by the immune system [32,33]. However, although Ipilimumab was shown to be effective against patients with unresectable stage III/IV metastatic melanoma [34] it was associated with significant adverse effects [35,36]. On the other hand, Nivolumab and Pembrolizumab have been approved for the treatment of unresectable and/or metastatic melanoma [27,37,38]. In addition, a combined protocol of Ipilimumab and Nivolumab is the main treatment option in patients with advanced BRAF-negative melanoma [39]. Finally, other types of immunotherapeutic agents/strategies include: (i) Interleukin-2 (IL-2), Interferon-a (IFN-a) and PEG/IFN-a (Pegylated/IFN-a), (ii) oncolytic viruses such as Talimogene Laherparapvec (T-VEC; to lyse tumor cells thus activating a non-specific immune response) and (iii) Adoptive Cell Transfer (ACT) therapies involving collection and modification of patient’s immune T cells [insertion of either chimeric antigen receptor (CAR) T cells and/or tumor-infiltrating lymphocytes (TILs)] which are then subsequently administrated to the patient for the enhancement of an immune response [4,32,40,41,42].

## 2 Epigenetics of Melanoma Development and Immune Resistance

**Figure 1:**
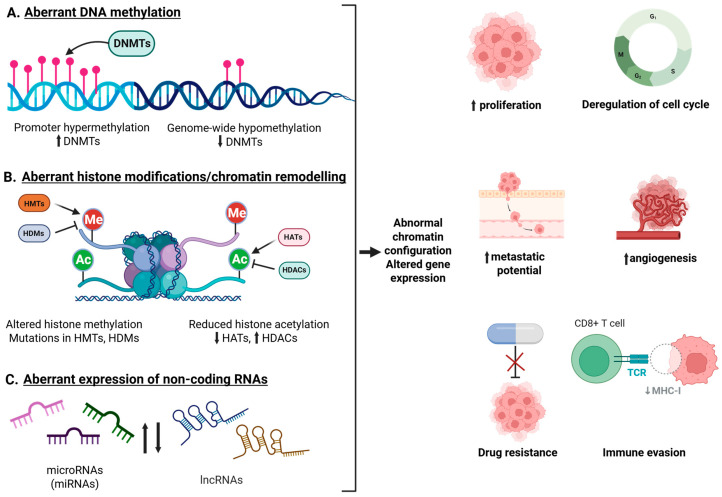
Epigenetic mechanisms involved in melanoma pathogenesis. Alterations of DNA methylation (hyper- and hypo-methylation), deregulated histone modifications/chromatin remodeling and altered expression patterns of non-coding RNAs (microRNAs/lncRNAs) are associated with melanoma’s pathophysiology. Specifically, an aberrant chromatin configuration/epigenetic landscape is responsible for the deregulation of gene expression patterns, involving silencing of tumor suppressor genes and/or activation of oncogenes, ultimately leading to melanoma progression through a variety of mechanisms including uncontrolled cell proliferation, deregulation of cell cycle progression, increased metastatic and angiogenic potential, as well as resistance to different therapeutic agents and ultimately evasion of immune responses. The figure was created with Biorender. DNMTs, DNA methyltransferases; HATs, histone acetyltransferases; HDACs, Histone deacetylases; HMTs, histone methyltransferases; HDMs, histone demethylases; lncRNAs, long-non-coding RNAs; TCR, T-cell receptor; MHC-I, Major histocompatibility Complex-I.

Epigenetics refers to heritable and reversible alterations of gene expression patterns, that in contrast to genetic mutations, are not associated with changes in the DNA sequence [43]. Generally, epigenetic mechanisms are categorized into three distinct classes: (i) DNA methylation, (ii) histone modifications/chromatin remodeling and (iii) alterations of non-coding RNA patterns. In this context, deregulation of the epigenetic landscape has been associated with the onset and development of various cancer types, including melanoma, through modulation of gene expression levels of oncogenes and/or tumor suppressor genes [44,45]. Specifically, various cellular processes contributing to proliferation, cell cycle progression, metastatic potential, angiogenesis, drug sensitivity or resistance, as well as immune-related functions and responses, are considered major factors in disease pathophysiology (Fig. 1). Given the reversible nature of epigenetic modifications, they appear as important therapeutic targets [46]. In this context, the development and use of “epi-drugs” either in mono- and/or combined treatment schemes with targeted therapies and immunomodulatory agents could result in substantial clinical benefit to melanoma patients [47].

### 2.1 DNA Methylation

DNA methylation is an important epigenetic mechanism that involves the addition of a methyl group at the fifth carbon in cytosine residues, an effect associated with stabilization of gene repression, especially when localized in proximity to transcription regions [48]. Such activity is mediated by specific enzymes called DNA methyltransferases (DNMTs) including DNMT1, DNMT3a and DNMT3b all of which utilize S-adenosyl methionine (SAM) as a methyl donor. DNMT1 is mainly associated with the maintenance of DNA methylation patterns during cell division while DNMTs3a & 3b maintain and establish *de novo* methylation in cytosine residues in both embryonic and somatic cells [48,49]. Generally, DNA methylation takes place in different regions across the genome called CpG dinucleotide islands found within gene promoters [50]. On the other hand, both hypermethylation of CpG islands as well as hypomethylation of repetitive elements and retrotransposons across the genome is associated with the oncogenic process. Such activity is mediated through a variety of mechanisms including chromatin remodeling, impairment of DNA repair mechanisms, genomic instability, alteration of signaling pathways, evasion of apoptosis, cell cycle deregulation, angiogenesis and metastatic potential, among others [51]. In this context, DNA hypermethylation has been described as an important epigenetic mechanism in melanoma development given that hypermethylation of tumor suppressor genes has been described extensively as a major epigenetic mechanism regulating melanoma pathogenesis. To this end, hypermethylation of more than 80 tumor suppressor genes has been described as a mechanism resulting in silencing of genes including: *CDKN2A*, *RASSF1A*, *TERT*, *MITF*, *p16INK4a/14ARF*, *PTEN*, *APAF-1* and *APC*, among others [43,46,52]. Accordingly, global DNA hypomethylation of genes such as *MAGE-A1/A2/A4/A6*, *GAGE 1-6*, *IL-2* and *LINE-1*, among others, has been described as a major epigenetic mechanism in melanoma progression associated with increased proliferative rates, angiogenesis, resistance to immunotherapies and an overall poor prognosis [44,45,53,54]. 

### 2.2 Histone Modifications

The basic functional unit of chromatin, nucleosome, consists of DNA wrapped around a histone complex (an octameric structure including two copies of H3, H4, H2A and H2B histone proteins) while N-terminal chains of histones protrude from the nucleosome. Those histone chains can be modified by different covalent post-translational modifications (PTMs) such as acetylation, methylation, phosphorylation, ubiquitylation, and sumoylation leading to chromatin conformational changes and ultimately modifications in gene transcription patterns [55]. Specifically, acetylation of histones is associated with a more relaxed chromatin configuration (euchromatin) allowing access to transcription factors thus favoring gene transcription. Such PTM is mediated by specific enzymes called histone acetyltransferases (HATs) while histone deacetylases (HDACs) remove acetyl groups thus causing chromatin compaction and transcriptional silencing [56]. On the other hand, histone methylation at lysine and arginine residues is another PTM mediated by histone methyltransferases (HMTs) that can mono-, di- and tri-methylate specific residues causing either transcriptional activation or repression. For instance, while trimethylation of lysine 9 and 27 residues of histone H3 (H3K9me3 and H3K27me3) and methylation of lysine 20 of histone H4 (H4K20) are related to transcriptional repression, methylation of lysines 4 (H3K4), 36 (H3K36) and 79 (H3K79) on histone H3 associate with transcriptional induction. On the contrary, histone demethylases (HDMs) remove methyl groups thus causing chromatin re-configuration [44,45,55,57]. In melanoma, different histone modifying enzymes such as HATs [KAT1/2B and CREB binding protein/p300 (CBP/p300)], HDACs [1/2/3/6/8 and Sirtuins (SIRTs) 1/2/3/6]; HMTs [SET Domain Bifurcated Histone Lysine Methyltransferase 1 (SETDB1), Histone-lysine N-methyltransferase 2D (MKT2D) and Enhancer of zeste homolog 2 (EZH2)]; and HDMs [Lysine-specific demethylase 1 (LSD1), Jumonji AT-rich interactive domain 1B (JARID1B), lysine (K)-specific demethylase 6B (KMD6B) and Jumonji domain-containing protein 2A (JMJD2A)] are known to become deregulated thus further contributing to melanoma’s increased invasiveness and metastatic potential, drug resistance, evasion of immunotherapy and overall poor prognosis [4,58,59].

### 2.3 Non-Coding RNAs (miRNAs/lncRNAs)

Non-coding RNAs are defined as untranslated transcripts capable of modulating gene expression patterns without changing the DNA backbone [60]. Generally, they are classified into two distinct classes: (i) small non-coding RNAs (miRNAs/microRNAs) of approximately 20–25 nucleotides and (ii) long non-coding RNAs (lncRNAs) with their size being over 200 nucleotides. Specifically, microRNAs can regulate translational processes through binding in their target mRNA transcripts at specific response elements and subsequently through the recruitment of RNA-induced silencing complexes which can alter/inhibit both stability and/or translation of their mRNA targets. In parallel, regulation of gene expression by lncRNAs is mediated through various processes including epigenetic regulation, genomic imprinting and chromosome dosage compensation [54,60,61]. On another note, both types of non-coding RNAs have been associated with important epigenetic mechanisms (e.g., DNA demethylation/transcription/imprinting, gene silencing and chromatin remodeling) while deregulated expression patterns have been implicated in the development of various cancers, including melanoma [62]. To this end, several miRNAs have been implicated in melanoma progression by means of evasion of apoptosis, uncontrolled proliferation, deregulation of cell cycle, metastatic and invasive potential as well as drug resistance. For example, down-regulation of miR-211, -196a, -21, -124, -29c and -210 has been reported in various stages of melanoma progression. On the contrary, over-expression of miR-210, -30b, -30, -149, -1908, -199a-5p and -199a-3p has been associated with immunosuppression, evasion of apoptosis and increased invasiveness and metastatic potential [63]. Finally, altered expression patterns of putative oncogenic lncRNAs [54] such as Survival Associated Mitochondrial Melanoma Specific Oncogenic Non-coding RNA (SAMMSON), Metastasis-Associated Lung Adenocarcinoma Transcript 1 (MALAT1), Antisense Noncoding RNA in the INK4 Locus (ANRIL), SPRY4 intronic transcript 1 (SPRY-IT1) and BRAF-activated non-protein coding RNA (BANCR) have been documented as important mediators of apoptotic evasion and acquisition of increased metastasis and invasiveness [54,64].

### 2.4 Immune Checkpoint Inhibitors and Immune Regulation in Melanoma

The development of targeted and immunotherapy-based approaches has revolutionized the therapeutic landscape in melanoma treatment. This is evidenced by doubling the 5-year relative survival rates in the last decades [65]. However, despite such significant progress, the 5-year overall survival rates for advanced-stage melanoma patients (treated with ICIs) are still around 50% [66]. Specifically, the use of different types of ICIs aims to stimulate immune responses against tumor cells through the inhibition/blockage of immune checkpoint molecules including the programmed cell-death protein 1(PD-1) and the cytotoxic T-lymphocyte-associated antigen 4 (CTLA-4). Both molecules are inhibitory receptors, expressed on activated effector T lymphocytes, that inhibit T-cell-mediated responses and prevent T-cell-induced autoimmune activity [47]. PD-1 is a glycoprotein expressed at the surface of T-lymphocytes or NKs, while its receptor [PD-1 ligand (PD-L1); CD274] is highly expressed on the surface of tumor cells. As such, PD-1/PD-L1 interactions can cause a negative signaling cascade leading to inhibition of CD8^+^ T-cell proliferation and/or cytokine secretion and inflammation thus inhibiting immune responses thereby resulting in the inhibition of tumor cells’ recognition and elimination. On the other hand, CTLA-4 is a cell surface marker that antagonizes the activity and binding of the lower-affinity co-stimulatory receptor CD28 to CD80 and CD86 on antigen-presenting cells (APCs), deactivating T cells (via IL-2 inhibition) and inhibiting cell-cycle progression [47,67]. In this context, the use of ICIs targeting PD-1/PD-L1 and/or CTLA-4, has been associated with significant clinical benefit in melanoma patients and thus considered as the main therapeutic option in advanced stages of the disease [60,68]. Specifically, more than half of melanoma patients treated with a combined scheme of Ipilimumab (an anti-CTLA-4 molecule) and Nivolumab (an anti-PD-1 molecule) had a significant and long-term clinical benefit. However, all remaining patients were characterized by a rapid progression of the disease due to ICI resistance [2,60,69]. Generally, ICI resistance is mediated by two distinct mechanisms: The so-called tumour-intrinsic ICI resistance that includes changes in DNA damage response, induction of specific signaling pathways and activation of cellular mechanisms of immune evasion; while the second one is relevant to tumour-extrinsic mechanisms that include tumor microenvironment (TME)-induced immunosuppressive alterations [60,70].

Cutaneous melanoma is characterized by high mutation rates and as such, it is considered one of the most immunogenic malignancies [71]. It is characterized by various immunosuppressive mechanisms [72] which favor evasion of both innate and adaptive immune mechanisms thus avoiding recognition and destruction by specific immune-related cells [e.g., dendritic cells (DCs), CD4^+^, CD8^+^ and natural killer (NKs) cells, among others] [71,73]. Generally, it has been described that malignant cells are characterized by different immunogenic characteristics, along their ability to produce several molecules with immune-modulatory properties ultimately leading to deregulation of immune cells’ activity as well as tumor immune infiltration [71]. In malignant melanoma, this is mainly attributed to the communication of melanoma cells with the surrounding TME. In addition, plasticity allows melanoma cells to adapt to the cytotoxic TME which could provide various neo-antigens thus favoring the attraction of immune surveillance cells and their elimination by T cells [71]. The architecture of TME is complex consisting of various components including cytokines, growth factors, the extracellular matrix (ECM), fibroblasts, myofibroblasts, adipocytes, keratinocytes, pericytes and different types of immune cells [4,74]. Cancer-associated fibroblasts (CAFs) have been shown to contribute to melanoma development through the interplay between melanoma cells and the surrounding TME leading to immune evasion and tumor adaptation [4,74]. Specifically, CAFs promote the phenotypic remodelling of melanoma cells into mesenchymal-like cells which in turn can activate phosphatidylinositol 3-kinases (PI3K) and thus induce resistance against various anticancer drugs [75]. On the other hand, since immune evasion is highly regulated through PD-1/PD-L1 interactions, tumor cells can activate myeloid-derived suppressor cells (MDSCs) that interact with CD8^+^ cells, through PD-L1, causing their inactivation by the secretion of both Transforming Growth Factor beta (TGF-β) and Interleukin-10 (IL-10) [76]. Consequently, the release of interferon-γ (IFN-γ), by CD4^+^ cells, leads to the activation of Tumour-Associated Macrophages (TAMs) also involved in the inactivation of CD8^+^ cells [77]. Moreover, melanoma cells can inhibit the cytotoxic activity of effector T cells through the recruitment of immunosuppressive regulatory T cells (Tregs) via secretion of chemokines [77].

### 2.5 Epigenetic Mechanisms of Immune Resistance in Melanoma

Analysis of clinical melanoma samples along with different melanoma cell lines, by utilizing genome-wide DNA methylation assays, revealed the inactivation of the cyclic GMP-AMP synthase (cGAS)/stimulator of interferon genes (STING) signaling pathway, through hypermethylation, and its implication in tumor antigenicity. However, that effect was reversed by the pharmacological inhibition of DNA methylation, resulting in restoration of the cGAS-STING signaling pathway, thus enhancing antigen presentation with a parallel promotion of T-cell mediated melanoma cell death [78]. In addition, another study reported that global DNA methylation levels directly affected PD-L1 expression levels, in melanoma, leading to evasion of immune responses. Such condition was reversed with Decitabine (a demethylating agent) which, in turn, induced PD-L1 expression levels [53]. On the other hand, methylation levels of the CTLA-4 promoter were found to be inversely associated with its expression levels as evidenced by a large cohort of malignant melanoma patients received anti-PD-1/PD-L1 immunotherapy. On the contrary, low levels of CTLA-4 promoter methylation were related to increased CTLA-4 expression levels, higher rates of immune activation/responses and overall increased survival rates among melanoma patients receiving anti-PD-1 or anti-CTLA-4 therapy. These results indicate its potential use as a biomarker in predicting immunological responses to both anti-PDL-1 and anti-CTLA-4 treatments [79]. Similarly, hypermethylation of *TNFRSF9* gene (a co-stimulatory receptor that enhances T-cell activation) was related to reduced responses against anti-PDL-1 treatments while hypomethylation of the same gene was shown to favor immune infiltration in the TME thus improving survival of melanoma patients [43,80]. In parallel, hypermethylation levels of *NLRC5* gene (a transcriptional regulator of MHC class I components such as HLA-A/C and B2M) were related with the suppression of those components. As a result, a compromised antigen presentation was observed ultimately leading to occurrence of resistance against ICIs, an effect associated with low survival rates among melanoma patients [81]. Overall, the above findings indicate that aberrant DNA methylation patterns reshape melanoma immunogenicity by silencing critical regulators of both antigen presentation and T-cell activation processes. In parallel, deregulated expression patterns of histone modifying enzymes have been also associated with resistance to immunotherapeutic approaches in malignant melanoma. For example, Histone Deacetylase 6 (HDAC6) promotes the expression of PD-L1 (through activation of the STAT3 pathway) which, in turn, establishes a self-reinforcing immunosuppressive loop that attenuates CTL cytotoxicity. In this context, pharmacological inhibition of HDAC6 appears as a therapeutic target by reversing resistance to ICIs [60,82]. In addition, histone demethylase LSD was responsible for the repression of interferon-stimulated genes as well as the antigen-presentation machinery. Accordingly, deficiency of this demethylase was associated with induction of tumor immunogenicity and T-cell infiltration ultimately leading to sensitization against anti-PD1 immunotherapy thereby promoting significant anti-tumor responses [4,60,83]. In the same context, nuclear localization/accumulation of phosphorylated LSD1was shown to be increased in PD-1^+^/CD8^+^ cells derived from resistant melanoma patients as well as in 4T1 immunotherapy-resistant mice, an effect linked to T-cell exhaustion. However, LSD1 blockade was efficient in reversing this effect [4,84]. On the other hand, up-regulated levels of Histone Methyltransferase EZH2, the catalytic component of the PRC2 complex, have been associated with aggressiveness, progression and poor prognosis among melanoma patients [85]. To this end, upregulated EZH2 expression levels were observed in a murine melanoma model following treatment with anti-CTLA-4 and IL-2 agents, leading to inhibition of important immune-related genes such as *TIM-3* and *LAG-3*. However, GSK503-mediated inhibition of EZH2 restored tumor immunogenicity, T-cell infiltration and interferon signaling and thus suppressing melanoma growth [4,59]. Similarly, EZH2 was reported to inhibit the expression of antitumor response related genes such as *RASSF5* and *ITGB2* both of which were implicated in DCs’ activation, CD4^+^ and CD8^+^ recruitment and tumor infiltration thus maintaining a non-tumorigenic microenvironment [86]. 

Interestingly, several studies have reported the involvement of various aberrantly expressed long noncoding RNAs and/or micro-RNAs in modulating immune responses in melanoma through post-transcriptional regulation of immune checkpoint mechanisms, interferon signaling and T-cell activation. For instance, deregulated expression of miR-17-5p, -25a/b, -28, -100, -222 as well as Melanoma Overexpressed (MELOE) were related to the occurrence of acquired resistance in melanoma following anti-PD-1 and anti-CTLA-4 treatments by sustaining PD-1/PD-L1 signaling and inhibiting T-cell immune activity [61,87]. On the contrary, expression levels of let-7e, miR-99b, -100, -125b, -146a, -146b and -155 were associated with resistance against ICI-induced treatments through activation of myeloid-derived suppressor cells (MDSCs) and induction of T-cell exhaustion thereby further contributing to resistance against immune checkpoint blockade [88]. Furthermore, up-regulated levels of miR-146a were responsible for immune system evasion, an activity mediated through negative regulation of the STAT-IFNγ axis and suppression of antigen-specific T-cell responses. In contrast, T cells derived from miR-146^−/−^ mice, were shown to possess higher expression levels of Stat1 (a miR-146a target gene) and IFNγ (a Stat1-regulated cytokine) [89]. In addition, different long-noncoding RNAs were shown to function as competing endogenous RNAs (ceRNAs) that sponge miRNAs targeting PD-L1thus favoring immune suppression. For example, NEAT1 has been reported to regulate PD-L1 expression leading to resistance against anti-PD-1/PD-L1 therapy [90]. On the other hand, emerging evidence also highlights exosomal lncRNAs as regulators of immune responses. For instance, LINC01214 released by melanoma cells, was responsible for the inhibition of IFN-γ, Granzyme-B, Perforin and TNF-α, by CD8^+^ cells, through up-regulation of the Phosphatase 1 Regulatory Inhibitor Subunit 11 (PPP1R11) via sponging miR-4492. The effect of LINC01214 was associated with resistance against PD-1 immunotherapy, in melanoma xenografts, while patients with low prognosis had higher expression levels of LINC01214, following PD-1 treatment, indicating its important role in altering immune responses [91].

Overall, an aberrant epigenetic landscape contributes to resistance against ICIs, in melanoma, by reducing tumor antigenicity and ultimately promoting an immune suppressive TME. Among deregulated epigenetic mechanisms, DNA hypermethylation of cGAS-STING, TNFRSF9/4-1BB and NLRC5 genes is associated with loss of MHC class I expression, reduced immune infiltration and activity of cytotoxic T lymphocytes as well as poor responses to anti-PD-1 therapy. Similarly, CTLA-4 promoter methylation patterns are inversely associated with its expression levels and is related with distinct clinical response rates. In this context, the above methylation-driven resistant responses appear as promising targets for DNMT inhibitors (DNMTis) such as 5-azacytidine, 5-aza-2′-deoxycytidine and Guadecitabine. On the other hand, deregulated expression of histone modifying enzymes (e.g., HDACs, LSD1/KDM1A, EZH2) have been associated with immunosuppressive responses and resistance including reduced antigen presentation as well as T-cell exhaustion and reduced infiltration. To this end, various epi-drugs such as HDAC6 selective inhibitors (e.g., Ricolinostat, Citarinostat, Nexturastat) and/or pan-HDACis (e.g., Entinostat, Panobinostat, Mocetinostat, Vorinostat) were shown to enhance the anti-melanoma effect of anti-PD1 and anti-CTLA-4 antibodies. Similarly, targeting EZH2 histone methyltransferase could restore interferon signalling immune infiltration as well as increase immune responses to ICIs. Moreover, deregulation of various microRNAs (e.g., miR-17-5p, -25a/b, -28, -100, -222, among others) and lncRNAs (e.g., NEAT1, LINC01214) sustain PD1/PD-L1 signaling, increase MDSC activity and leads to immune resistance responses against ICIs. In turn, their targeting through miRNA mimics, antagomirs and siRNAs, reverses resistance to immunotherapy. Finally, the use of BET bromodomain inhibitors (e.g., JQ1, IBET762, PLX51107, NHWD-870, BET151) is another approach for reversing resistance to ICIs through suppression of NF-κB as well as interferon-induced responsive gene programs, induction of intratumoral CD8^+^ T cells and enhancement of anti-PD-1 therapy.

## 3 Epigenetic Therapy and Melanoma Management

Based on the reversible nature of epigenetic modifications, the recent development of various epi-drugs can potentially reverse the aberrant epigenetic landscape thus restoring the expression of previously silenced genes involved in the pathogenesis of malignant melanoma. As such, the so called “epigenetic therapy” involves the use of drug compounds targeting a variety of deregulated epigenetic enzymes (e.g., DNMTs, HDACs, HATs, HMTs and HDMs) capable of inducing aberrant DNA methylation patterns as well as alterations in chromatin structure/remodeling contributing to melanoma development [4,44,46,47].

### 3.1 DNMT Inhibitors

Deregulated patterns of DNA methylation have been extensively described in contributing to carcinogenesis through silencing of tumor suppressor genes and evasion of various types of cell death [92]. In this context, the use of DNA methyltransferase inhibitors (DNMTis) appears as a promising approach in reversing aberrant DNA methylation thus restoring expression levels of previously silenced genes. DNMTis are nucleoside analogs capable of incorporating into DNA thus inhibiting the enzymatic activity of DNMTs and ultimately reversing gene silencing. The most used DNMTis include 5-azacytidine and 5-aza-deoxycytidine (Decitabine), an FDA approved therapeutic agent in patients with Chronic Myeloid Leukemia (CML), Myelodysplastic Syndrome (MDS) and Acute Myeloid Leukemia (AML) [93]. Additional types of nucleosides (e.g., Zebularine and Guadecitabine) and non-nucleoside (e.g., Asprocaine, MG98 and Hydralazine) DNMTis are currently being investigated for their anticancer activity in various other cancers [94]. In melanoma, the use of DNMTis as a therapeutic option has been utilized in combinatorial protocols with chemotherapeutic agents, other targeted drugs and/or various ICIs. For instance, melanoma cells treated with Decitabine and Carboplatin were shown to induce significant cytotoxicity and induction of apoptosis [95] while administration of Decitabine alone could reverse Temozolomide-induced resistance among melanoma patients in a phase I/II clinical trial [96]. Furthermore, a phase 1B study using combinatorial treatments including Vemurafenib and Decitabine resulted in reduced resistance rates and increased response duration among patients with metastatic melanoma [44,97]. On the other hand, Azacytidine has shown promising effects when combined with anti-PD-1 and anti-CTLA-4 antibodies as evidenced by induction of PD-L1 expression levels and an increase in tumor recognition and immune activity, by T-cells, respectively [53,85]. Finally, an increase of Human Leukocyte Antigen (HLA) class I expression was observed when melanoma patients, with unresectable III/IV stage, received a combined scheme of Guadecitabine and Ipilimumab [98].

### 3.2 Inhibitors of Histone Modifying Enzymes

Aberrations in histone acetylation and methylation contribute to melanoma progression through inhibition of anti-apoptotic genes, regulators of the PI3K/Akt signaling pathway and genes associated with cell cycle regulation and cellular development, among others [4,46,54]. Consequently, specific inhibitors of epigenetic enzymes have been developed among which HDACis [e.g., AR42, Valproic acid, Mocetinostat, Panobinostat, Vorinostat (SAHA), Trichostatin A (TSA), Entinostat, Nexturastat, Suprastat, ACY-241, Romidepsin, PCI-34051 and Tubastin] have been shown to inhibit HDACs implicated in melanoma progression in addition to sensitizing the action of various targeted drugs and immunotherapeutic agents [4,99]. 

For instance, exposure of melanoma cells to ACY1215 (Ricolinostat; an HDAC6i) caused induction of apoptosis and cell cycle arrest in G0/G1phase. In addition, combined treatments of Panobinostat and Encorafenib (BRAFi) caused a synergistic decrease in both RTK and PI3K signaling pathways in an *in vitro* model of melanoma. On the other hand, Entinostat (an HDAC3i) enhanced the anti-melanoma activity of different BRAF/MEKis in BRAF-mutant melanomas [100]. However, the main disadvantages of HDACis include high toxicity and non-selectivity profiles thus limiting their translation into clinical trials [51,99]. On the other hand, other classes of histone modifying inhibitors include GSK503 (an EZH2 HMTi) capable of reducing invasion and progression of melanoma, while combined inhibition of BRAF and EZH2 resulted in a synergistic anti-melanoma efficacy [43,101]. Finally, A485 (a potent and selective inhibitor of HMT p300/CBP) has been shown to possess significant anti-melanoma activity through increased cytotoxicity and promotion of cellular senescence in an MITF-dependent manner [102].

### 3.3 Inhibitors of Micro- and lncRNAs

Targeting aberrant ncRNA (micro- and lncRNAs) [103] expression patterns appears to be a promising therapeutic approach in melanoma management [87,103]. Indeed, restoration of either tumor-suppressor ncRNAs or inhibition of oncogenic ncRNAs (using miRNA mimics, antagomirs and anti-mRNA oligonucleotides) has been described as an efficient therapeutic strategy against melanoma. For instance, low levels of miR-200c have been described in BRAFi-resistant cell lines and clinical samples whereas restoration of their expression levels has been associated with impairment of resistance against various drugs [61,104]. In addition, encapsulated onco-suppressor miR-204-5p and miR-199b-5p (in lipid nanoparticles) reduced viability in an *in vitro* model of malignant melanoma [61,105] while R97/R98 (a chemically modified anti-miR-214 molecule) inhibited trans-endothelial migration and reduced the number of circulating tumor cells [106]. Accordingly, knockdown of lncRNA SPRY4-IT1 inhibited invasiveness and prevented tumor growth. In parallel, siRNA-mediated inhibition of hyper-expressed Urothelial Carcinoma Associated 1 (UCA1) and HOX Transcript Antisense Intergenic RNA (HOTAIR) lncRNAs resulted in significant reduction of motility and invasiveness of melanoma cells [107,108].

## 4 Targeting Epigenetic Mechanisms in Melanoma Immunotherapy

**Figure 2:**
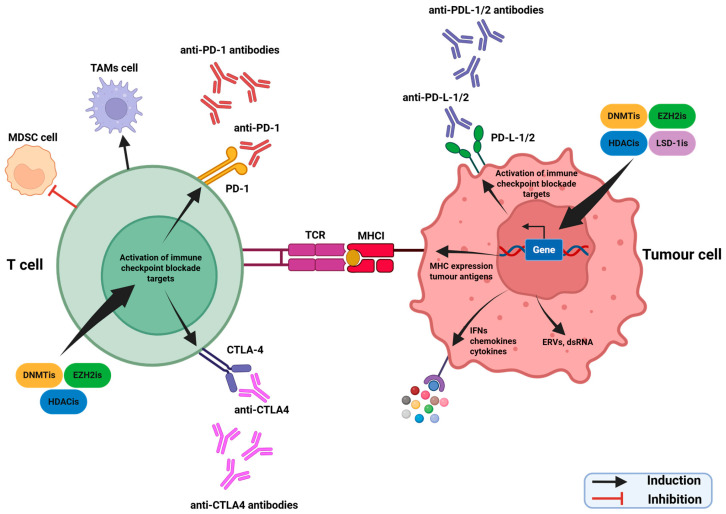
Targeting epigenetic mechanisms in regulating immune cell responses. Epigenetic drugs and immunotherapeutic agents have synergistic activity in regulating immunity of melanoma cells through different mechanisms including: (i) induction of ‘’viral mimicry’’ (ERV/dsRNA expression), (ii) activation of IFN responses, chemokines and cytokines, (iii) induction of antigen-presenting mechanisms, (iv) increase of tumour-associated antigens (v) reversal of T cell exhaustion, (vi) reprogramming of tumour-associated macrophages (TAMs) and myeloid-derived suppressor cells (MDSCs) towards pro-inflammatory states and (vii) activation of immune checkpoint blockade (PD1, PDL-1/2, CTLA-4). The figure was created with Biorender. TAMs, tumour-associated macrophages; MDSCs, myeloid-derived suppressor cells; DNMTis, DNA methyltransferase inhibitors; HDACis, Histone deacetylase inhibitors; EZH2is, Enhancer of Zeste homolog 2 inhibitors; LSD-1is, Lysine-(K)-specific demethylase 1A inhibitors; MHCI, major histocompatibility complex I; TCR, T-cell receptor; MHC-I, Major histocompatibility Complex-I; PD-1, programmed cell death protein 1; PD-L-1/2, programmed death-ligand 1/2, CTLA-4; cytotoxic T-lymphocyte-associated protein 4; IFNs, interferons; ERVs, endogenous retroviral elements; dsRNA, double-stranded RNA.

Although combinatorial schemes of targeted and immuno-based therapies are widely used for the treatment of metastatic melanoma, a major drawback is the development of drug resistance [4,38]. To this end, targeting the epigenetic landscape with epi-drugs appears to be a promising therapeutic option primarily through reversal of underlying immune resistance mechanisms. In the same context, several preclinical data have also provided a significant therapeutic benefit, following combinatorial protocols of epigenetic drugs and immunotherapy in regulating the anti-tumour immunity in melanoma [4,58,60]. Such effect is mediated through various mechanisms summarized in Fig. 2.

### 4.1 DNA Methyltransferase Inhibitors (DNMTis)

Aberrant DNA methylation patterns play a crucial role in cancer development and progression through modifications in the expression of various tumour suppressor genes [109]. For example, malignant melanoma is characterized by global hypomethylation marks as well as hypermethylation of CpG islands, both of which stimulate DNA instability and silencing of tumour suppression genes [110]. In turn, such alterations can activate various pathways, including apoptosis, cell-cycle growth arrest and DNA repair [15]. Interestingly, aberrant DNA methylation patterns have also been related to the regulation of immune-associated features of melanoma cells including antigen presentation, inflammation response and immune checkpoint molecules. 

In an *in vitro* melanoma model, treatment with the demethylating agent 5′-aza-2′-deoxycytidine enhanced the restoration of cell surface HLA class I and greatly potentiated the recognition of tumour cells by Melanoma Antigen Gene (MAGE)-specific cytotoxic T-lymphocytes (CTLs) [111]. Similarly, Guadecitabine promoted the upregulation of class I HLA antigens in addition to Intercellular Adhesion Molecule-1 (ICAM-1), thereby improving the therapeutic efficacy of Ipilimumab in an *in vitro* model of human malignant melanoma derived from surgically removed metastatic melanoma tumour specimens [112]. In another study5-azacytidine and 5-aza-2′-deoxycytidine were administered in a B16-F10 mouse melanoma model (along with an anti-CTLA-4 antibody) showing improved potency in the activity of anti-CTLA-4 [113]. In addition, inhibition of DNMTs triggered viral defence via the secretion of interferon beta 1 (*IFNβ1*) and the activation of a panel of Interferon Stimulated Genes (ISGs) such as *IF16*, *IFI27*, *IFI44*, *IFI44L*, *MX1*, *OASL* [113]. Beyond the effect of an aberrant DNA methylation pattern on tumour intrinsic pro-inflammatory pathways, regulation of various other immune checkpoint molecules (e.g., PD-L1, PD-L2, CTLA-4, LAG3, TIM-3 and Galectin-9) has also been reported [114]. Specifically, integrated genome analysis revealed that global DNA methylation induced the regulation of PD-L1 expression in melanoma [115]. Moreover, DNA methylation was also responsible for PD-L2 expression, thereby suggesting that the methylation status of PD-L2 can be used as a predictive biomarker for the progression of anti-PD-1-treated melanoma patients [116,117]. Finally, inhibition of DNA methylation can overcome immune checkpoint blockade. To this end, both 5-aza-5′deoxycytidine and Azacitidine have been reported to selectively bind and inhibit DNMT-1 and thus upregulate PD-L1 in melanoma cells [111]. 

Furthermore, treatment of B16-F10 melanoma cells with Guadecitabine has gained much attention as it was shown to stimulate the maturation of CD8^+^ T cells [117]. Moreover, combinational exposure of Guadecitabine with Ipilimumab led to a significant decrease in DNA methylation marks. This effect caused the upregulation of HLA class-I in melanoma cell lines and consequently induced the expression of CD8^+^ T and CD20^+^ B cells in post-treated tumours [117,118]. In another study, the combination of Guadecitabine with different ICIs [e.g., CD152 (an anti-CTLA-4) and CD279 (an anti-PD-1)] led to significant tumour reduction and metastatic capacity in a mouse B16-F10 melanoma model [119]. More specifically, Guadecitabine was shown to enhance the anticancer potency of ICIs through an increase of CD8^+^ T and NK cells [119] while this combination reduced the ability of tumour cells to infiltrate regulatory T-cells and MDSC in the TME [119]. Finally, increased levels of IFN-γ and IFN-γ-induced chemokines (with anti-angiogenic activity) were also reported [119].

### 4.2 Histone Deacetylase Inhibitors (HDACis)

Most reports investigating the effectiveness of HDACis in modulating tumour immunogenicity have focused on the expression of genes related to major histocompatibility complex (MHC) class-I or class-II antigen presentation. It was first reported that HDACis induced the expression of several proteins (e.g., TAP1, TAP2, LMP2, LMP7 and Tapasin) important for antigen processing and presentation in melanoma cells [120]. In addition, HDACs’ inhibition was shown to be strictly associated with the expression levels of ICIs, within the TME [121].

Moreover, HDACis have shown significant cytotoxicity against tumour cells beyond their ability to enhance immune-guided antitumor responses. For instance, Panobinostat, Entinostat and Mocetinostat induced the expression levels of PD-1, PD-L1 and PD-L2 in both *in vivo* and *in vitro* models, an effect attributed to an increase of histone acetylation marks at promoter regions of both *PD-L1* and *PD-L2* genes [121]. Specifically, increased histone acetylation mRNA levels of PD-L1 were correlated with its basal expression. To this end, upregulation of the expression of PD-L1 and PD-L2 was observed, in melanoma cells, which led to an increased therapeutic efficacy of the anti-PD-1 antibody [122,123].

On another note, HDAC6 [a member of the class-II HDAC superfamily which catalyses the deacetylation of Lys-72 residue of the extracellular signal-regulatory kinase 1 (ERK1)] appears to be highly expressed in various melanoma cell lines [124]. In addition, HDAC6 can bind to Tyrosine-protein phosphatase non-receptor type 1 (PTPN1), thus activating its downstream extracellular signal-regulated kinase 1/2 (ERK1/2) pathway, indispensable for melanoma progression. Another important function of a deregulated HDAC6 expression is the inhibition of apoptosis thus facilitating uncontrolled cell proliferation [125]. Interestingly, it has been reported that targeting HDAC6 was associated with an anti-melanoma effect characterized by impaired cell proliferation along with enhanced antitumor immunity (in terms of increased expression levels of MHC class-I and tumour-associated antigens) [126]. 

Pharmacological inhibition of HDAC6 appears to be an attractive approach in melanoma treatment. Indeed, the abolishment of HDAC6 activity was associated with alterations in gene expression levels of essential immune system modulators, including PD-1 and PD-L1 [127]. However, the selection of an appropriate HDAC6 inhibitor is of paramount importance, as administration of non-selective HDAC6 inhibitors can upregulate the expression of PD-L1, while selective inhibitors do not [128]. For instance, a recent study indicated an enhancement of immune system responses in patients with malignant melanoma upon administration of Ricolinostat and Citarinostat (selective HDAC6is) [129]. Their administration caused reduced production of ILs-4, -5, -6, -10 and -13, an effect accompanied by down-regulation of the transcription factor GATA3 and ultimately induction of T-Box Transcription Factor 21(T-BET). This effect was also accompanied by upregulation of central memory T-cells (e.g., CD45RA-CD45RO^+^, CD62L^+^, CCR7^+^) and a parallel decrease of T cell immunoglobulin and mucin-domain containing-3 (TIM3^+^), lymphocyte-activation gene 3 (LAG3^+^), PD1^+^ and Eomesodermin (EOMES^+^) PD1^+^ exhaustion-associated cells. Finally, a significant reduction of Forkhead Box P3 (FOXP3) expression levels resulted in suppression of T-regulatory cells (Tregs) [129]. On the other hand, Nexturastat (another selective HDAC6i) reduced the expression of PD-L1 in both *in vitro* and *in vivo* melanoma models as well as in clinical samples. This effect was mediated through alteration(s) in both recruitment and activation of signal transducer and activator of transcription 3 (STAT3) signalling pathway associated with major cellular processes (e.g., cell migration, proliferation and apoptosis) [130].

#### Combinatorial Studies of HDACis and Immune Checkpoint Inhibitors

The effect of immune checkpoint blockade is accompanied by the activation of myeloid-derived suppressor cells (MDSCs). To this end, Valproic acid (an HDAC inhibitor) together with an anti-PD-L1 antibody, induced the activation of IRF1/IRF8 transcriptional axis in MDSCs, thus inhibiting their function in a murine model of malignant melanoma. Specifically, blockage of the immunosuppressive function of MDSCs led to (i) decreased expression of IL-10, IL-6 and Arginase I (ARG1) as well as (ii) activation of CD8^+^ T-cells and ultimately secretion of Tumor Necrosis Factor a (TNFα) [131]. In another study, administration of Nexturastat A (a HDAC6 inhibitor), in a syngeneic melanoma model, improved the immune-efficacy of anti-PD-1 antibody and increased the number of (i) pro-tumorigenic M2 macrophages (TAMs), (ii) CD8^+^ T-cells and (iii) tumour-infiltrating NK cells [128].

At the molecular level, exposure to HDACis was associated with increased histone acetylation levels of *PD-L1* with a resultant durable and robust gene expression pattern. Moreover, the efficacy of combining HDACis (e.g., Mocetinostat, MS275, Belinostat, PCI34051 and Ricolinostat) with PD-1 blockade agents (Pembrolizumab or Nivolumab) was also evaluated in a B16-F10 murine melanoma model, indicating reduced rates of tumour progression and increased survival as opposed to the effect of single-agent treatment(s) [121]. On another note, the synergistic effect of HDACis and anti-PD-1 antibodies has been shown to induce immune cytotoxicity mediated through the activity of CD8^+^ T cells and NK cells in breast and pancreatic cancers [131,132]. Similarly, combined treatment of Entinostat with anti-PD-1agents promoted the activation of CD8^+^ T-cells, leading to tumour shrinkage in lung and bladder mouse models [133]. Furthermore, other studies have documented that Vorinostat and/or Entinostat induced the activation of NK-cells thus leading to cell lysis in advanced Non-Small Cell Lung Carcinoma (NSCLC) [134]. In addition, GC-745 (an HDACi) upregulated and potentiated the anti-cancer effect of the anti-PD-1 antibody through simultaneous activation of IL-2, IFN-γ expression and enhanced proliferation of cytotoxic T cells and NK-cells in various cancers [135]. In this context, pre-treatment of B16 mouse melanoma cells with two HDACis (e.g., AR42 and Sodium Valproate) potentiated the anti-cancer effect of both anti-PD1 and anti-CTLA-4 antibodies. Specifically, a significant increase in cytokine levels of CCL2, CCL5, CXCL9 and CXCL2 was observed, an effect associated with increased activation rates of T-cells, M1 macrophages, neutrophils and ultimately NK cell infiltration [136]. Overall, several reports have indicated the therapeutic efficacy of HDACis in enhancing the potency of ICIs through activation/potentiation of immune surveillance.

### 4.3 Bromodomain and Extra-Terminal (BET) Inhibitors

Another class of epigenetic molecules that modulate the expression of genes implicated in immune surveillance is the Bromodomain and Extra-Terminal domain (BET) proteins (e.g., BRD2, BRD3, BRD4 and BRDT) responsible for the recognition of histone acetylated lysine residues [47,137].

Through recruitment of a transcriptional machinery such as P-TEFb and RNA polymerase II, BET proteins sustain the expression of NF-κB–driven and interferon-responsive cascades [138,139], including the immune checkpoint ligand CD274 (PD-L1) [139]. BET inhibition (BETi; by JQ1 or I-BET762) suppresses BRD-dependent transcriptional activation [139,140], leading to downregulation of PD-L1 expression and other NF-κB targets in tumor cells [141]. This suppression alleviates immune evasion mechanisms, restoring CD8^+^ T-cell cytotoxicity, and enhancing anti-tumor immunity in multiple preclinical cancer models [141]. In this context, PLX51107 (a BETi) was tested for its effect on tumor growth in BRAF V600E melanoma syngeneic models and was shown to be capable of delaying melanoma tumor growth through activation and proliferation of CD8^+^ T cells. In addition, reduction of Cox2 PD-L1, FasL and IDO-1 expression was observed in the TME in parallel with an increased number of dendritic cells. Interestingly, PLX51107 was also efficient in delaying the growth of tumors under anti-PD-1 therapy, an effect accompanied by reduced expression of Cox2 and PD-L1on non-immune cells an induction of intratumoral CD8^+^ T cells [142]. Furthermore, another study revealed that a combination of NHWD-870 (a BETi) and Bacillus Calmette-Guerin vaccine was efficient in inhibiting tumor growth by enhancing T cells’ cytotoxicity. Specifically, MT1 was identified as a direct downstream target of BRD4 that was effectively suppressed by NHWD-870 in a humanized patient-derived xenograft model [142,143]. Finally, exposure of various malignant melanoma cell lines to BET151 (another BETi) was associated with inhibition of NF-κB activity and IFN-γ cytokine production leading to decreased expression of PD-L1 in melanoma cells [144]. Thus, BETis appear to act by reducing tumor-intrinsic immunosuppressive transcriptional programs including those regulated by NF-κB and MYC [4]. 

### 4.4 Lysine-Specific-Demethylase (LSD) Inhibitors

Lysine-(K)-specific demethylase 1A (LSD-1/KDM1A) is a specific histone demethylase catalysing the demethylation of lysine-3 (H3K4) and-9 (H3K9) residues in histone 3, at promoters and repetitive genomic elements, thereby silencing endogenous retroviral elements (ERVs) and antiviral genes [83]. LSD-1 is highly expressed in melanoma samples and is associated with resistance to anti-PD1 therapeutic approaches [47,137]. In this context, loss or inhibition of LSD-1 induces ERV transcription, resulting in accumulation of double-stranded RNA (dsRNA) and activation of a type I/II interferon (IFN) response, which allows antigen presentation (MHC-I) and chemokine expression (e.g., CXCL9, CXCL10), activated T-cells infiltration on tumours and potentiated the immunogenic activity of anti-PD-1 treatments, in a murine model of malignant melanoma [83]. These effects collectively convert immune-cold tumors into a more inflamed, antigenic phenotype, characterized by increased CD8^+^ T-cell infiltration and improved responsiveness to anti–PD-1 checkpoint blockade [83].

In addition, an inverse correlation between LSD-1 expression levels and CD8^+^ T cell infiltration rates was noted [83]. Moreover, PD-1^+^ CD8^+^ T cells derived from resistant melanoma patients expressed increased levels of nuclear LSD-1 phosphorylation at serine-111 (nLSD1p) [84]. As such, targeting the phosphorylated LSD-1 axis in the nucleus promoted the infiltration of IFN-γ/TNF-α-expressing CD8^+^ T cells within the tumour, an effect significantly enhanced when combined with immunotherapeutic agents, in immunotherapy-resistant melanoma-bearing mice [84]. Together, these findings illustrate a mechanistic contrast between BET and LSD1 inhibition: while BET inhibitors attenuate PD-L1/NF-κB–driven immunosuppressive transcription, LSD1 inhibition triggers ERV/dsRNA–mediated IFN signaling [83,145], resulting in distinct immunomodulatory and therapeutic outcomes [146]. Understanding these differences is critical for the rational design of combinatorial protocols and biomarker applications for immuno-oncology [84,146].

### 4.5 Enhancer of Zeste Homolog 2 (EZH2) Inhibitors

EZH2, a histone methyltransferase (HMT), is the catalytic subunit of the polycomb repressive complex 2 (PRC2). Specifically, EZH2 is responsible for chromatin compaction and gene repression through its methyltransferase activity via the SET domain [Su(var)3-9, Enhancer-of-zeste, Trithorax] by trimethylation of lysine (K)-27 residues at histone H3 (H3K27me3) [147]. Increased expression levels of EZH2 and H3K27me3 marks have been reported in various cancers, including melanoma [148]. Specifically, increased expression levels of both EZH2 and H3K27me3 marks were associated with silencing of tumour suppressor genes implicated in cellular proliferation and differentiation, along with modulation of immunological responses in malignant melanoma [148]. Particularly, EZH2 has been shown to modulate immune-editing and resistance to cancer immunotherapy due to T-cell infiltration and loss of antigen presentation, while its over-expression negatively influences the expression of interferon genes and T-helper cell type-1 (Th-1) chemokines in tumour cells. As such, pharmacological inhibitors of EZH2 can enhance tumour cell immunogenicity via activation of endogenous retroviruses and/or modulation of immune cell differentiation. For example, in a recent study, it was reported that inhibition of EZH2 (either by a short hairpin RNA or by the specific inhibitor GSK503) resulted in a loss of H3K27m3, an effect associated with the prevention of melanoma tumour metastasis [149]. Finally, EZH2 inhibition was shown to potentiate the immunotherapeutic potential of anti-CTLA-4 and IL-2 against an *in vivo* model of malignant melanoma [59].

### 4.6 Micro-RNAs (miRNAs)

MicroRNAs (miRNAs) are conserved small (21–25 nucleotides) non-coding RNAs that regulate gene expression and post transcriptional events. In this context, miRNAs can modulate the immune response in various cancers. For example, a recent study has suggested that miRNA-146a can act as a negative modulator of the immune system response, similar to that of ICP inhibitors [89]. miRNA-164a is a key pro-inflammatory regulator by affecting the Tumor Necrosis Factor Receptor Associated Factor 6-Tumor Necrosis Factor (TRAF6-TNF) axis in T-cells and the JAK/STAT/MHC axis in antigen-presenting cells [89]. In the context of the melanoma microenvironment, miRNA-164a was shown to act as a negative regulator of STAT1/IFN-γ, causing a decrease in IFN-γ levels, ultimately leading to a higher metastatic potential and reduced survival rates among melanoma patients [89]. Thus, inhibition of miRNA164a, by an antago-miR, increased IFN-γ expression levels, thereby suggesting that elevated IFN-γ stimulates the expression of PD-L1 in melanoma cells [89]. Accordingly, combinatorial treatment with miRNA-164a antagomir and anti-PD1was shown to be effective by increasing survival rates in a murine model of melanoma [89]. In parallel, it was shown that exhausted CD8^+^ T cells up-regulate multiple inhibitory receptors [e.g., PD1, TIM3 andB and T lymphocyte attenuator (BTLA)] whose expression levels correlate with T cell dysfunction [150]. In another study, melanoma tissues were shown to overexpress exhausted CD8^+^ T cells, with inhibitory receptors, which can modulate the expression of PD-1. To this end, 11 miRNAs were identified with significantly altered levels in the PD-1^+^ exhausted CD8^+^ T cells, of which miRNA-28 was reported to bind on multiple inhibitory receptors, thereby silencing PD-1^+^, decreasing the expression levels of PD-1, TIM3, LAG3 and BTLA in exhausted T cells and regulating PD-1^+^ Foxp3^+^ and TIM3^+^ Foxp3^+^ in exhaustive Treg cells *in vitro* [142]. In this context, treatment of a murine melanoma model with a miRNA-28 mimic was capable of recovering the ability of T-cells to secret cytokines, as evidenced by elevated expression of IL-2 and TNF-α [151].

On the other hand, long non-coding RNAs (lncRNAs) such as Metastasis Associated Lung Adenocarcinoma Transcript 1 (MALAT1) and Antisense Noncoding RNA in the INK4 Locus (ANRIL) are now recognized as regulators of chromatin architecture and post-transcriptional networks that determine tumour immunogenicity [152]. MALAT1 can modulate the serine/arginine (SR) splicing factor phosphorylation and distribution [153], a regulation that can trigger alternative splicing mechanisms which can modify the expression of immune-related genes, such as those encoding for cytokines and antigen-presentation molecules [154]. In addition, MALAT1 acts as a competing endogenous RNA (ceRNA) in several cancers (e.g., lung, prostate, breast, colorectal, liver, gastric, leukemia, brain and renal) thus functioning as a ‘molecular sponge’ for tumour-suppressive microRNAs [152,155]. Specifically, several studies have reported that MALAT1 indirectly regulates PD-L1 expression through miRNA-dependent circuits, including the MALAT1/miR-200a/PD-L1 and MALAT1/miR-195/PD-L1 axes [155], suggesting a mechanism by which MALAT1 can reduce antigenicity or chemokine secretion and thereby limit CD8^+^ T-cell recruitment [155,156]. Conversely, ANRIL has been shown to recruit Polycomb Repressive Complexes (PRC1 and PRC2) to specific genomic loci, resulting in H3K27me3 deposition and formation of repressive chromatin states that silences tumour-suppressor and immune-regulatory genes [157]. The recruitment of Polycomb complexes by ANRIL to gene promoters encoding for antigen-presentation molecules (e.g., MHC class I/II) and/or T-cell–attracting chemokines (e.g., CXCL9/10) is expected to reduce T-cell infiltration and promote an immune-cold tumour microenvironment (TME) [158]. Taken together, these mechanisms indicate how dysregulated expression patterns of lncRNA can collectively reprogram the tumour immune landscape and contribute to immune evasion in cancer [152].

## 5 Conclusions, Limitations and Future Directions

**Figure 3:**
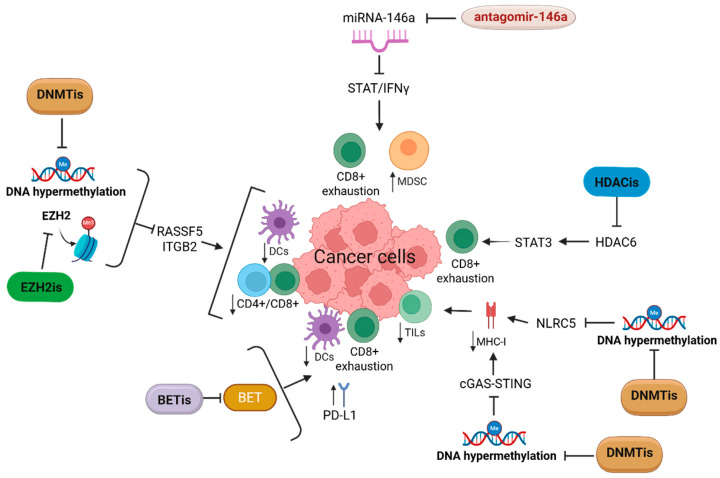
Epigenetic mechanisms in regulating immune cell responses and reversal of resistance. Aberrant epigenetic signatures affect tumour and immune cells, thus resulting in exhausted T cell phenotypes, alterations in PD-1/PD-L1 and CTLA-4 expression patterns and reduced antigen-presentation processes. For example, DNA methylation, posttranslational histone modifications and deregulated microRNA expression patterns can alter the expression of immune-related genes, activate immunosuppressive cells and signatures, thereby leading to antitumor immunity. As such, targeting epigenetic mechanisms with epi-drugs is an important therapeutic approach in reversing resistance. The figure was created with Biorender. MDSC, myeloid-derived suppressor cells; DCs, Dendritic cells; MHC-I, Major histocompatibility Complex-I; TILs, Tumor-Infiltrating Lymphocytes; DNMTis, DNA methyltransferase inhibitors; HDACis, Histone deacetylase inhibitors; BETis, Bromodomain and Extra-Terminal inhibitors.

In recent years, the use of immunotherapeutic agents has revolutionized the therapeutic approach to melanoma management. However, there are many melanoma patients who develop acquired drug resistance (especially in advanced stages of the disease), which is considered a major limitation towards prolonged survival rates. On the other hand, there is continuously growing evidence that deregulation of various epigenetic mechanisms (e.g., DNA methylation, histone modifications/chromatin remodeling and ncRNAs expression patterns) is associated not only with the pathophysiology of melanoma but also implicated with its resistance against targeted and immunotherapeutic drugs. Indeed, abnormal epigenetic signatures have been reported to affect the interaction between tumor and immune cells and thus can modulate the composition and role of the TME (Fig. 3). As such, epigenetic regulation appears to be a promising approach in treating melanoma by inducing a robust immune-based anti-tumor response. In this context, different epigenetic inhibitors, including those of DNMTis and histone-modifying enzymes, among others, have been utilized in various preclinical models of melanoma. Although there is strong evidence for their anti-melanoma efficacy, their translation into the clinical setting is rather limited, as evidenced by the lack of FDA-approved epi-drugs. However, most FDA-approved epi-drugs (DNMTis, HDACis and HMTis) are used as treatment options in several types of hematological malignancies, while only Tazemetostat (an EZH2 inhibitor) is currently used against solid tumors such as epithelioid sarcoma and follicular lymphoma. Specifically, the lack of clinical implementation of epi-drugs against melanoma is due to a variety of factors such as their high toxicity and low specificity, lack of pharmacodynamic characterization and limited development of biomarkers of therapeutic outcome. Moreover, these limitations significantly impede the selection of a rational dose and proper patient stratification to document a clinical benefit [4,46,47,159]. Indeed, the first-generation HDACis and DNMTis were developed and tested without any robust/standardized biomarkers that would predict and/or indicate their successful therapeutic index. In addition, side effects such as cardiac toxicity, cytopenia and myelosuppression have been documented to be indicative, at least partially, of their limited efficacy [160]. Moreover, epi-drugs have been commonly tested as sensitizers to various immunotherapeutic agents however, combinatorial exposures often result in cumulative toxicity, especially over prolonged duration(s) [161].

On the other hand, in pharmacodynamics, a major challenge is that a biologically effective dose of an epi-drug (either as a single agent and/or in combination with other immunotherapeutic agents) can potentially differ from the maximum tolerated dose(s). In turn, this could result in acute or chronic dose-limiting toxicities (DLTs). Since conventional phase I studies have been traditionally optimized based on acute pharmacotoxicity, they could be fundamentally misaligned with a pharmacodynamic effect based on chromatin modulation and remodeling, which can further indicate the requirement for prolonged assessment periods during clinical trials [162]. Specifically, in a recent trial (NIBIT-M4) evaluating the clinical efficacy of a combinatorial scheme with Guadecitabine and Ipilimumab, integrated analyses of paired pre- and on-treatment biopsies have established a connection between outcomes indicative of how tumor-tissue pharmacodynamic signatures can distinguish mechanisms of toxicity, thus enabling the identification of effective exposure protocols [163]. Similarly, there is significant scientific evidence that supports the use of circulating tumor DNA (ctDNA) as a prognostic, predictive and pharmacodynamic marker in metastatic melanoma, thereby offering a great opportunity in detecting either early responses and/or resistance signatures among melanoma patients under various treatment protocols [164]. Overall, before the development and clinical implementation of robust/specific biomarkers, various factors need to be taken into consideration, including that epigenetic endpoints are tissue-specific and assay-dependent. This is reflected in the fact that most candidate biomarkers have not yet been validated in the context of patients’ selection and stratification criteria, assessment of responses and outcomes and overall optimization of doses. Thus, improvements in trial design, selection of proper endpoints and dose administration are crucial for meeting the urgent need of prolonged assessment periods towards efficient and safe clinical applications [33,159]. 

Given the established role of an aberrant epigenetic landscape in ICI resistance, targeting specific epigenetic alterations appears to be a promising therapeutic approach. Various reports indicate the efficacy of different epi-drugs in combinatorial treatment protocols in both *in vitro* and *in vivo* models by reversing resistance through increased infiltration of CD8^+^ T cells, restoration of IFN-γ expression, increased tumor antigen presentation, modulation of the TME and overall promotion of anti-tumor immune responses [60]. In addition, data from clinical trials (using epi-drugs as single agents) were not associated with significant therapeutic efficacy. However, there are some promising clinical outcomes following combinatorial treatment protocols between various epigenetic inhibitors (mainly DNMTis and HDACis) and immunotherapeutic agents, especially ICIs (e.g., anti-CTLA-4 and/or anti-PD1 antibodies) (Table 1). For instance, in two clinical trials (e.g., NCT02608437 and NCT04250246), a combination of Decitabine and/or Guadecitabine (DNMTis) with ICIs was shown to have significant PFS and OS median rates (with limited or absent adverse effects) in patients with metastatic melanoma and anti-PD1/PDL-1 resistant melanoma. However, these promising results need to be evaluated in future clinical trials to validate the long-term survival rates of such combined protocols. On the other hand, in phase Ib of the NCT03565406 clinical trial, a combination of Mocetinostat (HDACi) with Ilipimumab and Nivolumab in patients with unresectable stage III/IV melanoma patients, showed almost 80% of ORR rates but only around 60% of patients experienced 3–4 grade adverse effects. On the other hand, in phase I of the NCT02032810 clinical trial a combination of Panobinostat (an HDACi) with Ipilimumab showed contradicting results in terms of ORR, mPFS and mOS in addition to being associated with severe adverse effects based on the concentration of Panobinostat used: causing either an immunosuppressive or an induction of pro-immunogenic response (when combined with ICI). Thus, there is an urgent need for a better clinical trial design in terms of selecting appropriate concentrations to reduce toxicity while developing robust and predictive biomarkers in evaluating clinical therapeutic efficacy [60].

Epigenetic therapy in melanoma is still at infancy because of the complexity of the epigenetic landscape. To this end, histone-modifying enzymes such as demethylase KDM5B can synergistically work with SETDB1 (an H3K9 methyltransferase) to silence immunogenic genomic elements [165] through the activation of the tumor-intrinsic antiviral/type-I IFN signaling that is required for effective T-cell priming and infiltration [166]. More specifically, KDM5 family members (e.g., KDM5A/KDM5B) were shown to suppress ERV expression and downstream dsRNA/IFN signaling by cooperating with KRAB-ZNF/KAP1 complexes and chromatin silencers (including recruitment of SETDB1 to retroelement loci), thus producing an immune-cold TME and resistance to immune mechanisms [165,167]. In parallel, *in vivo* studies have shown that loss of SETDB1 can suppress transposable elements, induce tumor-intrinsic type-I IFN responses and finally increase eMHC-I and chemokine expression levels. Such suppression stimulates production of CD8^+^ cells indicating the involvement of SETDB1 in immune evasion in melanoma models [166]. Overall, the KDM5B–SETDB1 pathway acts as a common mechanism of immune evasion and targeting this pathway, by inhibiting KDM5B, SETDB1 and/or their regulators (e.g., USP7), can potentially restore IFN signaling, increase antigen presentation and thus make tumors more responsive to ICIs [168,169]. These new insights highlight promising directions of future clinical trials focusing on the evaluation of combinatorial schemes using novel epigenetic inhibitors with various ICIs.

On the other hand, recent developments in emerging technologies such as single cell epigenomics and spatial transcriptomics can provide a significant in-depth examination of melanoma’s TME. Specifically, these technologies revealed a high degree of epigenetic heterogeneity within tumor cells, adjacent stroma and immune cells [170,171]. Distinct and different patterns of chromatin organization and accessibility coexist within spatially defined tumor niches, ultimately resulting in differentially altered gene expression profiles. Such heterogeneity can shape specific transcriptional programs associated with evasion of immune system responses, T-cell exhaustion and resistance to ICIs [172]. In melanoma, such epigenetic heterogeneity is responsible for the alteration of antigen presentation, interferon signaling pathway, as well as the expression of immunosuppressive ligands, thereby influencing tumor responses to immunotherapeutic agents. Importantly, spatial transcriptomics integrates such epigenetically induced transcriptional states within spatially defined tumor regions, thus enabling the identification of specific areas and cell types potentially associated with immune resistance. As such, identification and mapping of the above mentioned different epigenetic states within the TME appears as a main approach for the application of more precise anticancer approaches through the combination of epigenetic drugs with immunotherapeutic agents (to reprogram resistant tumor and/or immune cells) for a more efficient anti-melanoma effect [173,174]. 

In addition, epigenetic priming has been described as a significant strategy to improve CAR-T cell therapy by means of increasing tumor cell vulnerability to cytotoxic lymphocytes and limiting the rates of immune T cells’ exhaustion [175]. For example, pre-treatment of B-cell lymphoma with DAC was shown to increase CD19 antigen expression while enhancing the activity of CAR-T therapy, as evidenced by complete remission (CR) of patients [176]. Moreover, Decitabine-treated chimeric antigen receptor T (dCAR-T) cells exhibited significant anti-tumour activities and increased cytokine production and proliferation rates in both *in vitro* and *in vivo* models. Such effect was mediated through increased expression levels of proliferation-, memory- and cytokine production-associated genes in dCAR-T cells. Interestingly, it was reported that tumour-infiltrating dCAR-T cells were characterized by a relatively high expression of memory-related genes as opposed to those with low expression of exhaustion-related genes in an *in vivo* model [177]. Finally, M344 (a class I HDACi) and Chidamide could enhance CAR-T cell function, in both *in vitro* and *in vivo* models, by increasing memory maintenance and resistance to exhaustion of CAR-T cells. This was mediated through inhibition of HDAC1 expression and an increase of H3K27ac marks, leading to the activation of the canonical Wnt/b-catenin signaling pathway [178]. Although studies combining epigenetic priming with CAR-T cell therapy in melanoma are relatively rare, inhibition of DNMTs with DAC (a demethylating agent) was efficient in upregulating expression of HLA class I and ICAM-1 in melanoma cells while improving their recognition by gp-100 cytotoxic T lymphocytes (CTL) [179]. Furthermore, combined treatments of DAC and IFN-I inhibited the growth of melanoma both *in vitro* and *in vivo* through induction of *Mx1* (an IFN-stimulated gene), activation of adaptive immune responses and promotion of CD8^+^ T cells while reducing the homing of immunosuppressive CD11b+ myeloid cells and regulatory T cells [180]. Moreover, in a recent study utilizing a 3D surfaceome screen in primary melanoma cultures, Decitabine induced the expression of ICAM-1 and HLA class I/II, along with the secretion of pro-inflammatory cytokines CXCL9/10, an activity associated with increased immunogenicity and significant immune cell infiltration [181]. Overall, there is strong evidence that epigenetic priming with the use of specific epi-drugs (e.g., DNMTis, HDACis) could enhance CAR-T cell therapy and thus appear to be a promising therapeutic approach in melanoma management. 

Finally, recent studies indicate the association and influence of microbial metabolites against epigenetic mechanisms and overall epigenetic reprogramming. In the context of cancer progression and therapeutic response to different classes of epi-drugs, tumor-associated dysbiosis has been described as an important mechanism in altering the methylation landscape in liver and colon cancers. Accordingly, microbiome-derived metabolites can act as epigenetic modifiers through changes in DNA methylation patterns and histone modifications, thus altering chromatin configuration and inducing transcriptional reprogramming in TME. Specifically, these metabolites can alter (through epigenetically induced mechanisms) the function and expression of antigen-presenting cells and tumor-infiltrating lymphocytes [182]. For instance, short-chain fatty acids (SCFAs) produced by bacterial fermentation were capable of inhibiting HDAC enzymes, altering histone acetylation marks and inducing extrathymic differentiation of Treg cells in mice, while treatment of naïve T cells with microbe-derived butyrate increased the acetylation levels in the promoter of *Foxp3*, indicating a potential mechanism underlining the regulation of Treg cells’ differentiation [183]. In addition, analysis of baseline stool samples obtained from patients with metastatic melanoma, prior to immunotherapy, revealed a link between commensal microbiome and clinical responses. Reconstitution of germ-free mice with fecal material, from responding patients, caused an increase in T cell responses an effect associated with improvement of the anti-PDL-1 therapy [184]. A recent clinical trial investigated if modifications of gut microbiota could reverse the resistance to anti-PD-1 treatments [through responder-derived fecal microbiota transplantation (FMT) along with anti-PD-1] in patients with PD-1-refractory melanoma. Data indicated that this approach reversed resistance in a subgroup of PD-1 advanced melanoma patients through alterations of gut microbiome and reprogramming of TME [185]. Collectively, these results highlight the potential benefit of microbiome-related interventions in re-sensitizing cancer cells to epigenetic-based interventions as single agents as well as in combinatorial treatment protocols utilizing different ICIs. Stratification and/or identification of specific microbiome/metabolite profiles that produce distinct epigenetic signatures, among patients, would be of great importance in future studies for the rational selection of specific epi-drugs and ICIs, for the improvement of therapeutic outcome(s).

Overall, a better understanding of the complexity of the underlying molecular mechanisms regulating epigenetic alterations involved in melanoma pathophysiology will allow us to develop more efficient and highly selective epi-drugs with fewer off-target effects thus limiting toxicity and favoring increased antitumor responses. Currently, there are several trials investigating into the efficacy of novel epigenetic therapies towards new targets (e.g., LSD1, BETis) in various cancers. At the same time, the direct involvement of epigenetic mechanisms in regulating the TME further support the use of treatment protocols utilizing different epi-drugs combined with ICIs to enhance their therapeutic outcome and/or reverse immune resistance responses (Table 2). In the context of melanoma’s therapeutic management, development of robust biomarkers together with prolonged assessment periods and improvements in trial design, selection of proper end points and dose administration will potentially translate into a more robust clinical benefit among melanoma patients.

**Table 1:** Clinical trials of combinatorial treatments of epigenetic drugs with ICP inhibitor-based therapies for the treatment of malignant melanoma.

Drug Intervention	Condition	Status	Phase	Identifier
Guadecitabine (DNMTi) Ipilimumab (anti-CTLA-4 mAb)	Unresectable or metastatic Melanoma	Unknown	Phase1	NCT02608437
Guadecitabine (DNMTi) Nivolumab (anti-PD-1 mAb) Ipilimumab (anti-CTLA-4 mAb)	Melanoma Non-small Cell Lung Cancer	Currently not recruiting	Phase 2	NCT04250246
Decitabine (DNMTi) Peg-Interferon	Melanoma	Terminated	Phase 1	NCT00791271
Panobinostat (HDAC) Ipilimumab (anti-CTLA-4 mAb)	Melanoma Skin Neoplasms	Active, not recruiting	Phase 1	NCT02032810
Entinostat (HDACi) Pembrolizumab (anti-PD-1 mAb)	Melanoma	Recruiting	Phase 2	NCT03765229
Tinostamustine (HDACi) Nivolumab (anti-PD-1 mAb)	Malignant Melanoma	Recruiting	Phase 1	NCT03903458
Azacitidine (DNMTi) Recombinant interferon alfa-2b	Recurrent Melanoma Recurrent Renal Cell Cancer Stage III Melanoma Stage IV Melanoma Stage IV Renal Cell Cancer	Completed	Phase 1	NCT00217542
Azacitidine (DNMTi) Pembrolizumab (anti-PD-1 mAb)	Melanoma and Other Malignant Neoplasms of Skin Metastatic Melanoma	Active, not recruiting	Phase 2	NCT02816021
Mocetinostat (HDACi) Induction Phase + Ipilimumab (anti-CTLA-4 mAb) + Nivolumab (anti-PD-1 mAb) Mocetinostat (HDACi) Maintenance Phase + Ipilimumab (anti-CTLA-4 mAb) + Nivolumab (anti-PD-1 mAb)	Melanoma	Terminated	Phase 1	NCT03565406
HBI-8000 (HDACi) Nivolumab (anti-PD-1 mAb)	Melanoma Non-Small Cell Lung Cancer Renal Cell Carcinoma	Active, not recruiting	Phase 1 Phase 2	NCT02718066
4SC-202 (Domatinostat) (HDACi) Pembrolizumab (anti-PD-1 mAb)	Malignant Melanoma	Completed	Phase 1 Phase 2	NCT03278665
Abexinostat (HDACi) Pembrolizumab (anti-PD-1 mAb)	Stage III Cutaneous Melanoma Stage IV Cutaneous Melanoma Locally Advanced Melanoma Locally Advanced Solid Neoplasm Metastatic Head and Neck Squamous Cell Carcinoma Metastatic Malignant Solid Neoplasm Metastatic Melanoma Metastatic Urothelial Carcinoma Non-Small Cell Lung Carcinoma Stage IB Lung Cancer Stage III Cutaneous Squamous Cell Carcinoma of the Head and Neck Stage III Lung Cancer Stage III Ureter Cancer Stage IIIA Lung Cancer Stage IIIB Lung Cancer Stage IIIC Lung Cancer Stage IV Cutaneous Squamous Cell Carcinoma of the Head and Neck Stage IV Lung Cancer Stage IV Ureter Cancer Stage IVA Lung Cancer Stage IVB Lung Cancer	Active, not recruiting	Phase 1	NCT03590054
Entinostat (HDACi) Pembrolizumab (anti-PD-1 mAb)	Non-Small Cell Lung Cancer Melanoma Mismatch Repair-Proficient Colorectal Cancer	Active, not recruiting	Phase 1 Phase 2	NCT02437136
Azacitidine (DNMTi) Pembrolizumab (anti-PD-1 mAb)	Melanoma and Other Malignant Neoplasms of Skin Metastatic Melanoma	Active, not recruiting	Phase 2	NCT02816021
CPI-1205 (Lirametostat) (EZH2i) Ipilimumab (anti-CTLA-4 mAb)	Advanced Solid Tumours	Completed	Phase 1	NCT03525795

**Table 2:** Summary of combinatorial protocols of different classes of epi-drugs with immunotherapeutic agents, describing their mechanism of action, clinical status and synergies with immunotherapy.

Class	Mechanism of Action	Representative Agents/Clinical Status	Synergy with Immunotherapy	Ref.
DNMTis	Inhibit DNA methyltransferases leading either to global or local DNA hypomethylation; Re-expression of silenced genes (including endogenous retroviruses and tumour antigens)	Azacitidine Decitabine (approved in hematologic malignancies) Guadecitabine (in trial)	Induce “viral mimicry” (ERV/dsRNA expression); activates innate dsRNA sensors and type I IFN signaling; upregulate antigen-processing and chemokines secretion leading to an increase in T-cell recruitment and improved responsiveness to ICI in preclinical/early clinical studies	[186,187]
HDACis	Inhibit histone deacetylases leading to reduction in histone acetylation associated with a compact chromatin structure (heterochromatin) related to transcriptional inactivation	Vorinostat Panobinostat Entinostat (approved in some hematologic/solid tumor settings or in trial. It is also being tested in combination with anti-PD-1/PD-L1)	Reprograms tumour-associated macrophages (TAMs) and myeloid-derived suppressor cells (MDSCs) towards pro-inflammatory states; enhances tumour antigen expression, chemokine-driven T-cell infiltration and lower immunosuppressive cell functions; Synergy with anti-PD-1/PD-L1 in preclinical/early clinical combinations	[188,189]
EZH2is	Inhibit EZH2 histone methyltransferase which results in decreased levels of trimethylated lysine 27 residues on histone 3, thus leading to de-repression of silenced loci	Tazemetostat (approved for epithelioid sarcoma and some lymphomas)	Increases tumour immunogenicity by activating antigen-presentation/chemokine genes and overcoming T-cell exclusion; Preclinical and emerging clinical trials test EZH2i in combination with ICI inhibitors to improve T-cell infiltration and CAR-T efficacy	[190,191]
BETis (bromodomain inhibitors)	Inhibit BET proteins (BRDs 2/3/4) from binding to acetylated histones thus decreasing transcriptional activity at key enhancers/promoters including inflammatory and checkpoint-related genes	JQ1 I-BET family (in clinical trials) PLX51107 (in clinical trials)	Suppresses NF-κB/BRD4-driven PD-L1 and inflammatory/myeloid programs in tumour and stromal cells; reduces adaptive immune suppression and promotes CD8^+^ T-cell–mediated tumour control; rationale for combination with ICIs where BETi reduce tumour-intrinsic immunosuppression	[192,193]
LSD1 (KDM1A) inhibitors	Inhibits H3K4/H3K9 demethylase activity of LSD; Associates with repressive chromatin states; activates repetitive elements and IFN-stimulated genes	Seclidemstat (SP-2577) (in clinical trials and preclinical studies)	Activates endogenous retroviral elements and induces IFN response; increases histocompatibility complex class I (MHC-I), chemokine expression and CD8^+^ T-cell infiltration; synergy with anti-PD-1 supports clinical evaluation of combinatorial protocols	[194,195]
DNMTis + HDACis (combinatorial)	Combination of DNA hypomethylation and increased histone acetylation induces activation of previously silenced genomic regions	Dual agents in combinatorial protocols (clinical development)	Induction of viral mimicry and innate immune activation; enhancement of antigen presentation and chemokine secretion allowing T-cell recruitment; enhanced ICIs responses in preclinical models	[196,197]

## Data Availability

Not applicable.

## Abbreviations

ACT	Adoptive Cell Transfer
AML	Acute Myeloid Leukemia
ANRIL	Antisense Noncoding RNA in the INK4 Locus
APCs	Antigen-presenting cells
ARG1	Arginase-1
BANCR	BRAF-activated non-protein coding RNA
BET	Bromo- and Extra-Terminal domain
BRAF	V-Raf Murine Sarcoma Viral Oncogene Homolog B
BTLA	B- and T-lymphocyte attenuator
CAFs	Cancer Associated Fibroblasts
CAR T	Chimeric Antigen Receptor T cells
cGAS	Cyclic GMP-AMP synthase
CML	Chronic Myeloid Leukemia
CTLs	Cytotoxic T-lymphocytes
CTLA-4	Cytotoxic T Lymphocyte-associated Antigen-4
DITC	Dacarbazine
CBP/p300	CREB binding protein/p300
DNMT	DNA methyltransferase
ECM	Extracellular Matrix
EOMES	Eomesodermin
ERK1	Extracellular signal-regulated kinase-1
ERVs	Endogenous retroviruses
EZH2	Enhanced of Zeste Homolog 2
FOXP3	Forkhead box P3
HAT	Histone acetyltransferase
H3K27me3	Trimethylation of Histone H3 at Lysine 27
H3K4	Histone H3 lysine 4
H3K9	Histone H3 lysine 9
H3K36	Histone H3 lysine 36
H3K79	Histone H4 lysine 79
H3K9me3	Trimethylation of Histone H3 at Lysine 9
H3K27me3	Trimethylation of Histone H3 at Lysine 27
H4K20	Histone H4 lysine 20
HDAC	Histone deacetylase
HDM	Histone demethylase
HLA	Human leukocyte antigen
HMT	Histone methyltransferase
HOTAIR	HOX Transcript Antisense Intergenic RNA
ICAM-1	Intercellular adhesion molecule 1
ICIs	Immune Checkpoint Inhibitors
IFN-*n*	Interferon
IL-*n*	Interleukin
ISGs	Interferon Stimulated Genes
JAK/STAT	Janus kinase-signal transducer and activator of transcription
JARID1B	Jumonji AT-rich interactive domain 1B
JMJD2A	Jumonji domain-containing protein 2A
JNK	Jun N-terminal kinase
KDM6B	lysine (K)-specific demethylase 6B
LAG3^+^	Lymphocyte-activation gene 3
LDH	Lactate dehydrogenase
LMP*n*	Latent membrane protein
lncRNAs	Long non-coding RNAs
LSD1	Lysine-specific demethylase 1
MAGE	Melanoma Antigen Gene
MALAT1	Metastasis-Associated Lung Adenocarcinoma Transcript 1
MDS	Myelodysplastic syndrome
MDSCs	Myeloid-Derived Suppressor Cells
MELOE	Melanoma Overexpressed
MHC	Major histocompatibility complex
miRNAs/microRNAs	Small non-coding RNAs
MITF	Microphthalmia-associated transcription factor
MKT2D	Histone-lysine N-methyltransferase 2D
NK	Natural Killer
NSCLC	Non-Small Cell Lung Carcinoma
nLSD1p	Nuclear LSD1 phosphorylated at serine 111
PD-1	Programmed cell death protein 1
PD-L1	Programmed death-ligand 1
PFS	Progression-free survival
PI3K/Akt	phosphatidylinositol 3-kinase/Protein kinase B
PRC2	Polycomb repressive complex 2
PTM	Post-translational modification
PTPN1	Protein Tyrosine Phosphatase Non-Receptor Type 1
SAMMSON	Survival Associated Mitochondrial Melanoma Specific Oncogenic Non-coding RNA
SETDB1	SET Domain Bifurcated Histone Lysine Methyltransferase 1
SIRTs	Sirtuins
SPRY4-IT1	SPRY4 Intronic Transcript 1
STAT	Signal transducer and activator of transcription
STING	Stimulator of interferon genes
TAMs	Tumour-associated macrophages
TAP*n*	Transporter associated with antigen processing
T-BET	T-Box Transcription Factor 21
TGF-β	Transforming growth factor beta
Th-1	T-helper cell type-1
TILS	Tumor-Infiltrating Lymphocytes
TIM3^+^	T cell immunoglobulin and mucin-domain containing-3
TME	Tumour microenvironment
TNF-α	Tumour necrosis factor alpha
TRAF6-TNF	Tumor Necrosis Factor Receptor Associated Factor 6-Tumor Necrosis Factor
Tregs	Regulatory T cells
TSA	Trichostatin
UCA1	Urothelial Carcinoma Associated 1
